# Application of density clustering with noise combined with particle swarm optimization in UWB indoor positioning

**DOI:** 10.1038/s41598-024-63358-4

**Published:** 2024-06-07

**Authors:** Hua Guo, Haozhou Yin, Shanshan Song, Xiuwei Zhu, Daokuan Ren

**Affiliations:** School of Electronic and Information Engineering, Shandong University of Science and Technology, Qingdao, 266590 China

**Keywords:** Electrical and electronic engineering, Computer science

## Abstract

Due to the presence of non-line-of-sight (NLOS) obstacles, the localization accuracy in ultra-wideband (UWB) wireless indoor localization systems is typically substantially lower. To minimize the influence of these environmental factors and improve the accuracy of indoor wireless positioning, this paper proposes a density clustering with noise combined with particle swarm optimization (DCNPSO) to improve UWB positioning. Which exploits the advantages of the density-based spatial clustering algorithm with noise (DBSCAN) and particle swarm optimization (PSO) algorithm. The experimental results show that the DCNPSO algorithm achieves 45.25% and 36.14% higher average positioning accuracy than the DBSCAN and PSO algorithms, respectively. The positioning error of this algorithm remains stable within 3 cm in static positioning and can achieve high accuracy in NLOS environments.

## Introduction

UWB, an emerging high-precision positioning technology, has developed rapidly in recent years with continuously improving accuracy and significantly reduced costs^1^. Compared with other positioning technologies, UWB technology has obvious advantages, such as strong anti-interference ability and good penetrability, making it particularly suitable for indoor positioning^2^. It is gradually applied in related fields such as smart elderly care, intelligent manufacturing, tunnel transportation, and warehousing logistics^3,4^. However, the needed positioning precision is frequently not met due to the influence of numerous impediments in indoor environments^5^. Therefore, it is vital to minimize or eliminate the influence of such errors.

The Density-Based Spatial Clustering of Applications with Noise (DBSCAN) algorithm is capable of dividing regions with a high density of points into clusters while filtering out regions with a low density of points, ultimately obtaining clusters of arbitrary shapes in the data set with noise^6,7^. The DBSCAN algorithm is not sensitive to the sequence of object inputs or impacted by noise, and it can locate clusters of different sizes and shapes in addition to reliably identifying outliers. In UWB indoor positioning, obstacles and NLOS environments often introduce noise interference into static positioning data, resulting in increased errors that impact the accuracy of positioning^8^. The aforementioned advantages of the DBSCAN algorithm make it suitable for UWB positioning data processing, as it effectively filters out noise and narrows down the accuracy range for subsequent steps.

Particle swarm optimization (PSO) algorithm based on the theory of group intelligence, through the cooperation and competition between the particles in the group generated by the group intelligence optimization search^9^. The particle swarm optimization algorithm possesses traits that allow particles to be influenced not only by their own evolution but also to learn and remember the collective evolution within groups. This enables the particles to achieve optimal adjustments autonomously. In the global model, only the globally optimal particle can provide information to other particles, and the entire search process follows the current optimal solution. As a result, all particles converge quickly towards the optimal solution. This algorithm has attracted attention for its advantages of easy implementation, high accuracy, and fast convergence, and is superior in optimizing the problem of UWB indoor positioning accuracy^10^.

When using UWB for indoor positioning, it is affected by NLOS errors and multipath interference. These interferences introduce a large amount of random noise in the positioning process, which affects the positioning results of UWB. To mitigate the impact of random errors on UWB indoor positioning, this paper proposes a density clustering with noise combined with particle swarm optimization (DCNPSO) by combining the advantages of DBSCAN and PSO. The algorithm utilizes clustering to deal with noise and combines the optimization-seeking ability of particle swarm to achieve accurate positioning.

The reminder of this paper is organized as follows. In “Related work” section introduces the current research status of clustering algorithm and particle swarm optimization. In “Preliminary concepts”, introduces the principle of double-sided two-way ranging and the algorithms of DBSCAN and PSO. In “UWB positioning based on trilateral barycentric algorithm and DCNPSO algorithm” section, we introduce the innovative content of the paper, the trilateral barycentric algorithm and the DCNPSO algorithm in detail. In “Measured data and result analysis” section, we apply the proposed strategies to UWB indoor positioning scenarios and evaluate the DCNPSO algorithm and its application in UWB indoor positioning through a large number of experiments. Finally, a conclusion is given in the last section.

## Related work

### UWB positioning based on clustering algorithm

Arsan et al.^11^ proposed the positioning optimization algorithm based on the fusion of a self-organizing mapping (SOM) neural network and an unscented Kalman filtering algorithm. As a result, the average localization error is reduced by 43.26%. Luo et al.^12^ propose a new trilateration algorithm based on the combination and K-Means clustering to effectively remove the positioning results with significant errors in this paper, which makes full use of the position and distance information of the anchor nodes in the area. However, trilateral localization algorithms often do not accurately localize to a point. Wang et al.^13^ processed the parameters and extracted useful multipath information through a joint clustering algorithm improved by the k-means clustering algorithm and the mean shift clustering algorithm. However, this algorithm cannot solve the positioning problem in nonlinear systems.

### UWB positioning based on PSO algorithm

Yun et al.^14^ propose an improved particle swarm optimization (PSO) process to design a high-performance compact ultrawideband (UWB) filter. However, if it is used for indoor positioning, its positioning accuracy and stability will be greatly reduced. Cai et al.^15^ proposed ELPSO algorithm, three different kinds of PSO variants, namely global PSO (GPSO), local PSO (LPSO), and bare bones PSO (BBPSO), are hybridized to complement each other. And it improves the accuracy and reliability of UWB positioning. Lakshmi et al.^16^ propose an algorithm that incorporates ELPSO and PSO-BPNN for UWB localization based on^15^. The algorithm simulation has a significant positioning effect with an average error of 2.7 cm, but it has not been verified by real experiments.

## Preliminary concepts

The steps of UWB wireless indoor positioning to obtain the tag position are as follows: The first step is to calculate the distance and other parameters from the signal received by the receiver, namely ranging^17^. The second step is to use the acquired parameters to determine the position of the target point according to the relevant algorithm, namely positioning^18^. The UWB positioning method used in this paper is the combination of the DS-TWR ranging algorithm and the trilateral barycentric algorithm for positioning.

### Double-sided two-way ranging

**Figure 1:**
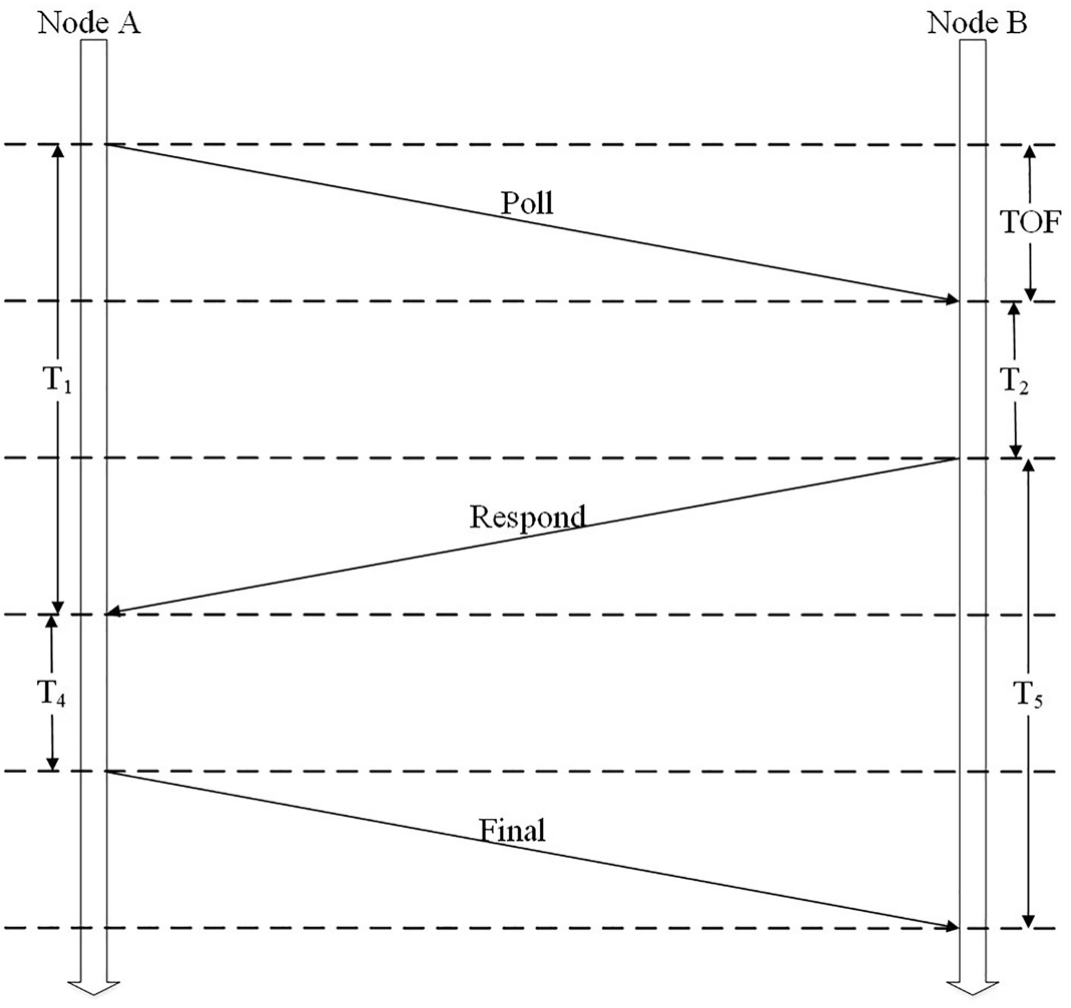
Double-sided two-way ranging schematic diagram.

Double-sided two-way ranging (DS-TWR) is a technique that builds on the fundamentals of single-sided two-way ranging^19^. It reduces inaccuracies even in response delays that are quite lengthy by combining two round-trip time measurements to produce flight time results^20^. The ranging principle of the DS-TWR algorithm is shown in Fig. 1. Node A sends a ranging packet 1 Poll signal to node B, and after $T_2$ time, node B returns a ranging packet 2 Respond signal. Node A sends another Final signal to node B after $T_4$ time until node B receives the signal. The flight time between node A and node B is obtained, and the distance between the nodes is calculated by multiplying the flight time by the speed.

From the Fig. 1 we can get:

1 $$\begin{aligned} {T_1}= & {} 2 \times TOF + {T_2} \end{aligned}$$

2 $$\begin{aligned} {T_5}= & {} 2 \times TOF + {T_4} \end{aligned}$$

3 $$\begin{aligned} {T_1} + {T_4}= & {} {T_2} + {T_5} \end{aligned}$$

This is obtained by multiplying the left and right sides of 1 and 2 and shifting the terms:

4 $$\begin{aligned} {T_1} \times {T_5} - {T_2} \times {T_4} = 2 \times TOF \times ({T_5} + {T_2}) \end{aligned}$$

The signal time-of-flight (TOF) is obtained by combining 3 and 4:

5 $$\begin{aligned} TOF = \frac{{{T_1} \times {T_5} - {T_2} \times {T_4}}}{{{T_1} + {T_2} + {T_4} + {T_5}}} \end{aligned}$$

The formula of DS-TWR ranging error calculation is:

6 $$\begin{aligned} {TOF_{error}}=TOF\times \left( 1-\frac{{k_a + k_b}}{2}\right) \end{aligned}$$

where $k_a$ and $k_b$ denote the frequencies of device A and device B, respectively.

The DS-TWR method mitigates the impact caused by different crystal oscillation offset errors in various devices and enhances ranging accuracy^21^. Therefore, the UWB ranging method in this experiment adopts DS-TWR.

### Density-based spatial clustering of applications with noise

The DBSCAN algorithm is a density-based clustering algorithm that automatically identifies different clusters and separates them from noisy data^22^. The algorithm works by first identifying the core points by calculating the number of points within the Eps neighborhood of each point. Then, for each core point, the algorithm searches for all points that are connected to its density to form clusters. Finally, points that are not labeled as core or boundary points are labeled as noise points. The processing flow of the DBSCAN algorithm is as follows:

1. Arbitrarily select a data object point p from the data set;
2. The *Eps* neighborhood and the minimum number of neighborhood points, *MinPts*, for point p are calculated. If point p is classified as a core point based on the given parameters *Eps* and *MinPts*, it will be used to identify and form a cluster consisting of all closely connected data points^23^;
3. If the selected data object point p is an boundary point, select another data object point;
4. Repeat steps 2 and 3 until all points are processed.

### Particle swarm optimization

The particle swarm optimization algorithm is an idea inspired by the study of the bird predation problem^24^. The PSO algorithm initially initializes a swarm of particles in the feasible solution space, where each particle represents a potential optimal solution to the optimization problem. The characteristics of each particle are represented by three indicators: position, velocity, and fitness value.

7 $$\begin{aligned} {\left\{ \begin{array}{ll} X_i=(x_{i1},x_{i2},\cdots ,x_{id})\\ V_i=(v_{i1},v_{i2},\cdots ,v_{id})\\ Pbest_{id}=(pbest_{i1},pbest_{i2},\cdots ,pbest_{id})\\ Gbest_{d}=(gbest_{1},gbest_{2},\cdots ,gbest_{d}) \end{array}\right. } \end{aligned}$$

Where $X_i$ represents the position of the particle. $V_i$ represents the velocity of the particle. *Pbest* is the best position that the particle has searched for. *Gbest* is the best position that the population has experienced.

The particle moves in the solution space and updates the individual position by tracking the individual pole *Pbest*^25^ and group pole *Gbest*^26^. For each updated position of the particle, the fitness value is calculated and the individual pole *Pbest* and population pole *Gbest* positions are updated by comparing the fitness value of the new particle with the fitness values of the individual pole and population pole.

1. Initialize the population with information about each particle including random position and random velocity.
2. According to the objective function, calculate the fitness value of each particle.
3. For each particle, its current fitness value is compared to the fitness value corresponding to the best historical position (*Pbest*). If the current fitness value is higher, the current position is updated with the *Pbest*.
4. For each particle, its current fitness value is compared to the fitness value corresponding to the group’s historical best position (*Gbest*). If the current fitness value is higher, the current position is updated with the *Gbest*.
5. Judge whether the search results meet the stop condition (reach the maximum number of iterations or meet the accuracy requirements). Output the ideal value if the stop condition is met; if not, return to Step 3 and keep running until the condition is met.

## UWB positioning based on trilateral barycentric algorithm and DCNPSO algorithm

### Trilateral barycentric algorithm

**Figure 2:**
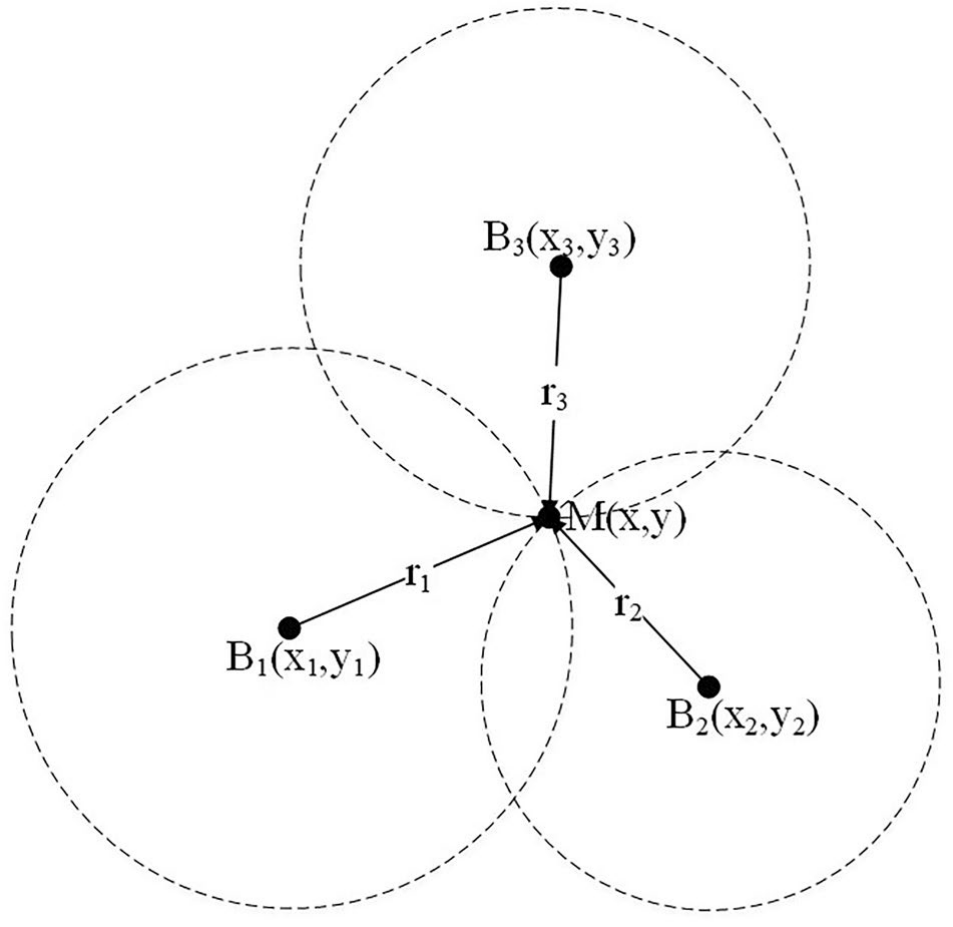
Trilateral algorithm.

The trilateral algorithm involves solving the positioning coordinates of the tag from the distance value obtained from DS-TWR ranging. In the ideal situation, the ranging circles drawn from the distance values will intersect at one point, as shown in Fig. 2$B_1$, $B_2$, and $B_3$ represent the base stations, and M represents the tag. Assuming that the mobile tag M’s coordinates are (*x*, *y*) and that the base station positions are $B_1$($x_1$, $y_1$), $B_2$($x_2$, $y_2$), and $B_3$($x_3$, $y_3$), respectively, and that the distances between each base station and the mobile tag are represented by the letters $r_1$,$r_2$, $r_3$.

**Figure 3:**
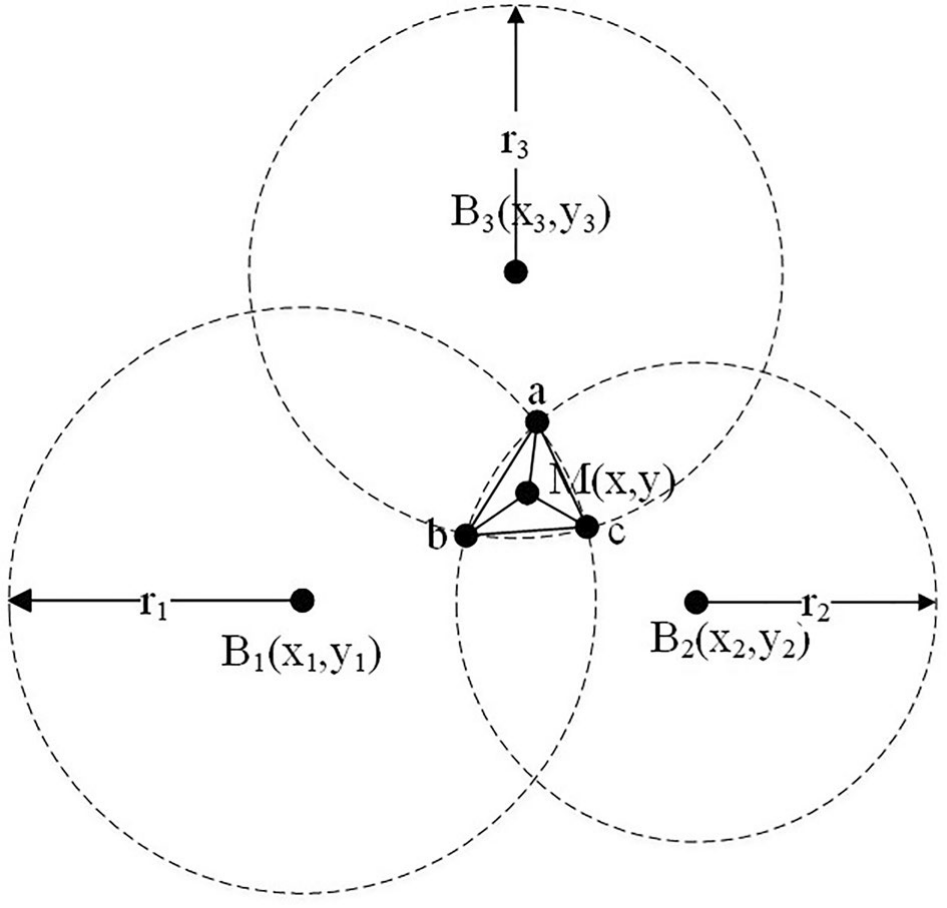
Trilateral barycentric algorithm.

However, the distance value measured in the actual situation is not accurate, which leads to the three ranging circles intersecting at three points to form a region. As shown in Fig. 3. This paper proposes a Triangular Barycentric Algorithm, which uses the centroid M of the triangle formed by connecting the intersection points a, b, and c of three circles as the tag positioning point.

The equations of the three ranging circles are:

8 $$\begin{aligned} \left\{ \begin{array}{l} {(x - {x_1})^2} + {(y - {y_1})^2} = r_1^2\\ {(x - {x_2})^2} + {(y - {y_2})^2} = r_2^2\\ {(x - {x_3})^2} + {(y - {y_3})^2} = r_3^2 \end{array} \right. \end{aligned}$$

By expanding 8, there can be:

9 $$\begin{aligned} \left[ \begin{array}{l} - 2{x_1}\;\; - 2{y_1}\;\;1\\ - 2{x_2}\;\; - 2{y_2}\;\;1\\ - 2{x_3}\;\; - 2{y_3}\;\;1 \end{array} \right] \left[ \begin{array}{l} \;\;\;\;x\\ \;\;\;\;y\\ {x^2} + {y^2} \end{array} \right] = \left[ \begin{array}{l} r_1^2 - x_1^2 - y_1^2\\ r_2^2 - x_2^2 - y_2^2\\ r_3^2 - x_3^2 - y_3^2 \end{array} \right] \ \end{aligned}$$

The 9 corresponds to the linear equation $AX=B$, and the error can be reduced by solving the M coordinate using the least squares method. The obtained (x,y) represents the final positioning coordinate point of the tag. The specific result is as follows:

10 $$\begin{aligned} X = {({A^T}A)^{ - 1}}{A^T}B \end{aligned}$$

### UWB positioning based on the DCNPSO algorithm

**Figure 4:**
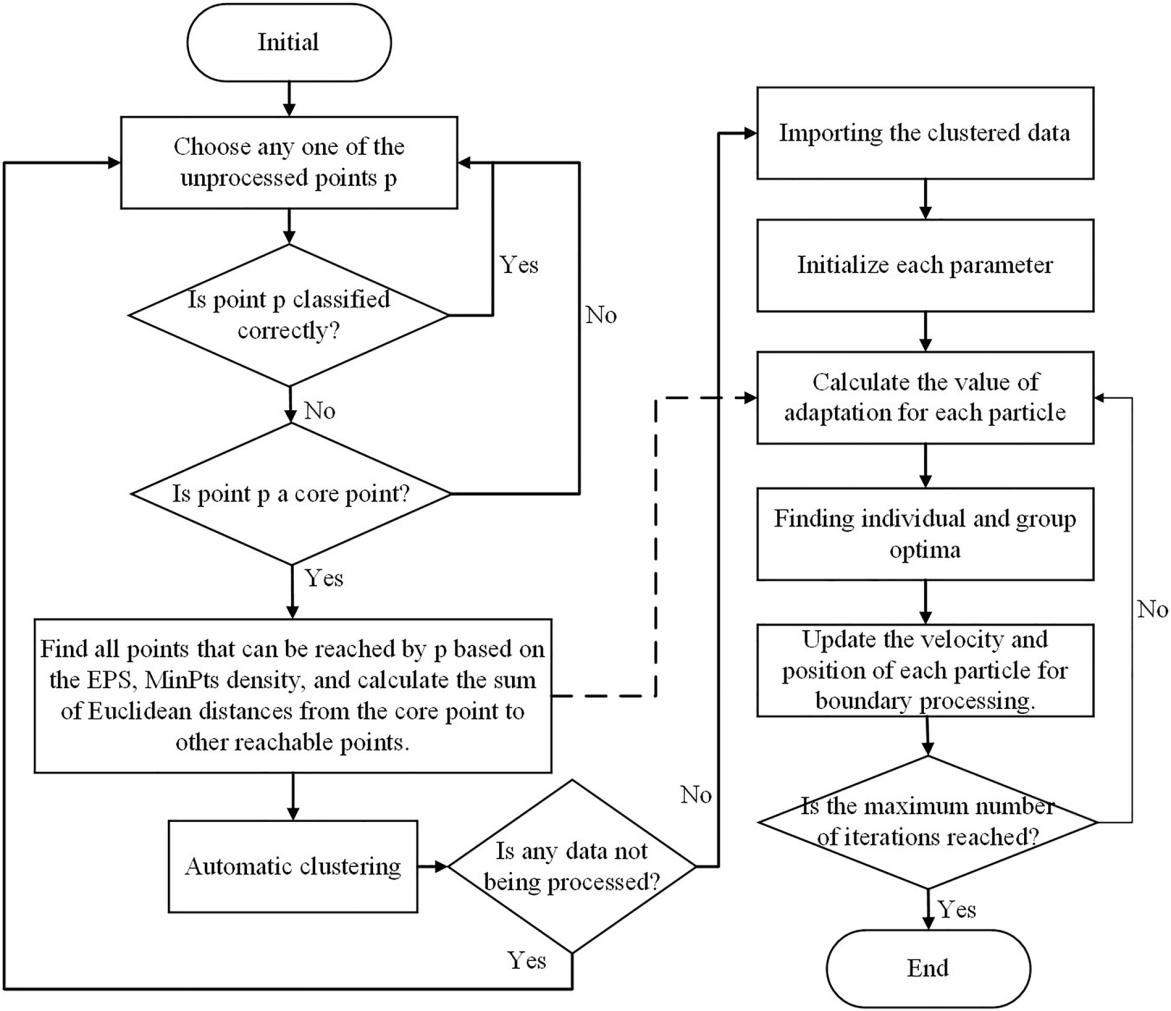
Flowchart of DCNPSO algorithm.

The basic idea of density clustering with noise combined with particle swarm optimization (DCNPSO) is as follows: Firstly, the DBSCAN algorithm is applied to UWB positioning data. Second, the effective cluster data is retained while filtering out noisy cluster data. Finally, particle swarm optimization is applied to optimize the retained effective cluster results and update the fitness. The minimum sum of the Euclidean distance between the core point and other points calculated by DBSCAN is taken as the fitness of the PSO algorithm, and the optimal solution is found through continuous iteration. The DBSCAN algorithm can filter out noisy interfering data after processing, and the PSO algorithm can globally optimize the processed data, thereby improving the accuracy of UWB positioning. The flowchart of this algorithm is shown in Fig. 4. The processing flow of the DCNPSO algorithm is as follows:

1. Initializes the control parameters. Including sample set size, individual historical optimal position *Pbest*, group global optimal position *Gbest*, position limit range *Xlimit*, speed limit range *Vlimit*, iteration number *T*=1000, set evolutionary algebraic counter *t*=0, neighborhood radius $Eps=0.75$ and minimum neighborhood number $MinPts=30$.
2. For the original UWB positioning data, the DBSCAN algorithm is applied to the data according to *Eps* and *MinPts*.
3. Normalization of data processing.
4. The sum of Euclidean distance between the core point and other points in the second step of the calculation process is taken as the fitness of the particle and calculates the fitness value of each particle.
  11 $$\begin{aligned} Fitness=\sum _{i=1}^{n}\sqrt{\left( x_{i}-x_{f}\right) ^{2}+\left( y_{i}-y_{f}\right) ^{2}} \end{aligned}$$
  Where $(x_f,y_f)$ are the core point coordinates.
5. Update the particle state based on the velocity and position update formulas, calculate the fitness value of each particle after the update, compare the optimal fitness value of each particle with its fitness value at the historical optimal position and the population optimal value, and replace it if it is better. And store the individual and group optimal positions along with their corresponding optimal fitness values. Speed Updates:
  12 $$\begin{aligned} {v^{k + 1}} = w{v^k} + {c_1}rand_1^k(pbest - {x^k}) + {c_2}rand_2^k(gbest - {x^k}) \end{aligned}$$
  Where *w* is the inertia weight, which is set to 0.5. $c_1$, and $c_2$ are the learning factors, which take a fixed value of 2. $rand_1^k$ and $rand_2^k$ are two random numbers on the interval [0,1]. Location Updates:
  13 $$\begin{aligned} {x^{k + 1}} = {x^k} + {v^{k + 1}} \end{aligned}$$
6. Boundary handling. During the iteration process, if a particle’s position exceeds the boundaries of position and velocity, the position and velocity values of the particle are set to the boundary values.
7. Determines whether the search results meet the termination condition (to reach the maximum number of iterations T or to meet the accuracy requirements). If the stop condition is satisfied, the optimal value will be outputted and the algorithm will end; Otherwise, go back to step 5 and continue running until the condition is satisfied.

## Measured data and result analysis

### Data collection

**Figure 5:**
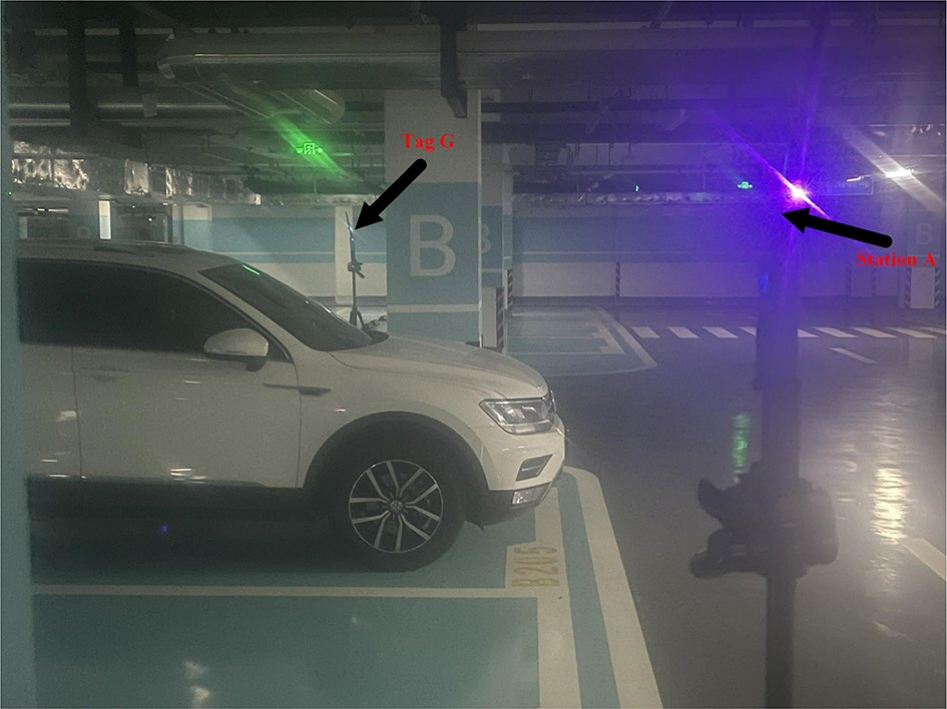
Actual photo of the garage.

**Figure 6:**
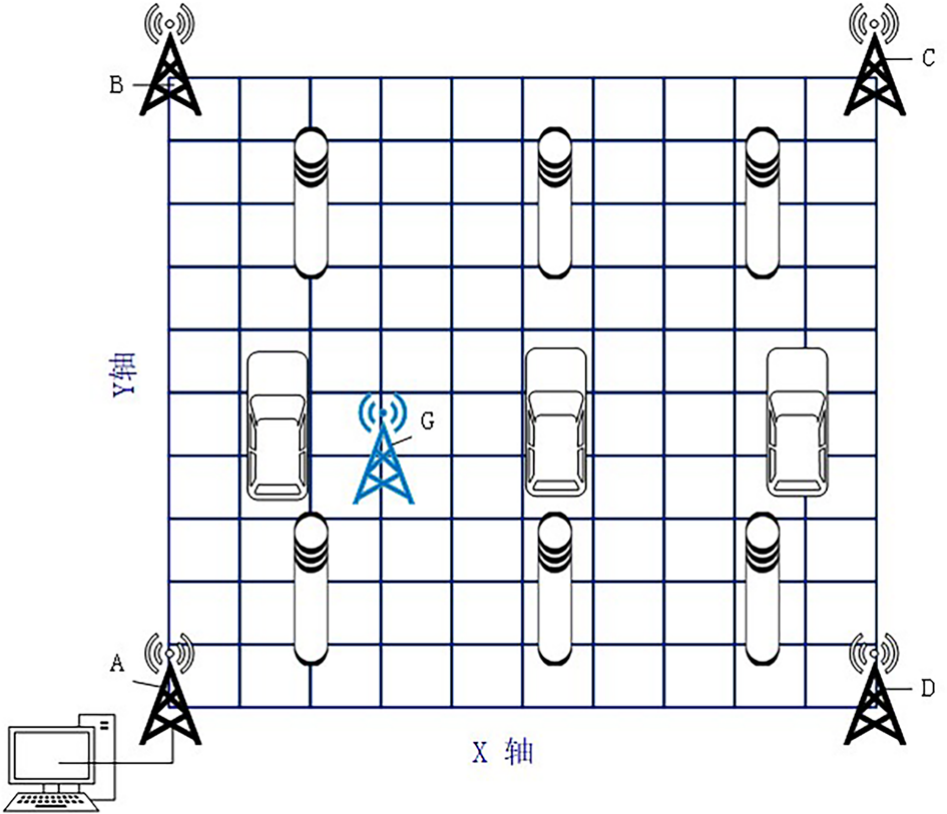
Simulation of the experimental site.

The experimental hardware in this paper consists of UWB positioning module components and a host PC. During the experiments, we used a stand with a height of about 1.5 meters to fix the base station and tag. The remaining four base stations are also positioned at the same height and fixed to the brackets located at the corners of the square. The experimental site is situated in an underground parking garage, as depicted in Fig. 5. In this environment, there are NLOS interferences such as cars and load-bearing columns, which meet the requirements of an indoor setting. To simplify the calculations, we select base station A as the main localization base station and connect it to the computer via the serial port. Taking the main base station A as the origin of the coordinate axis o, the X-axis is defined along the straight line where AD is located, and the Y-axis is defined along the straight line where AB is located. The plane right-angle coordinate system is established in meters. The sub-base stations B, C, and D are positioned at the coordinates (0.40), (40.40), and (40.0) respectively. In the experiment, the x and y coordinates of the localization tag G are separately analyzed, as shown in Fig. 6. By analyzing this data, we can evaluate the accuracy of the positioning and perform subsequent processing and optimization.

**Figure 7:**
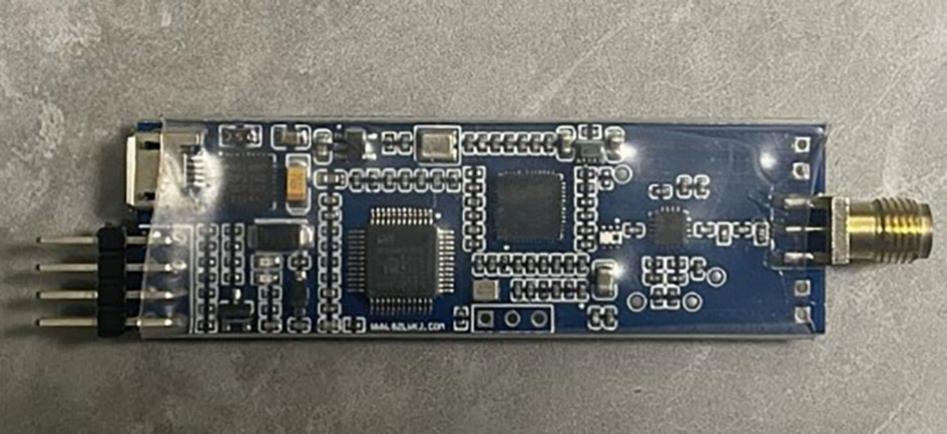
UWB module.

**Figure 8:**
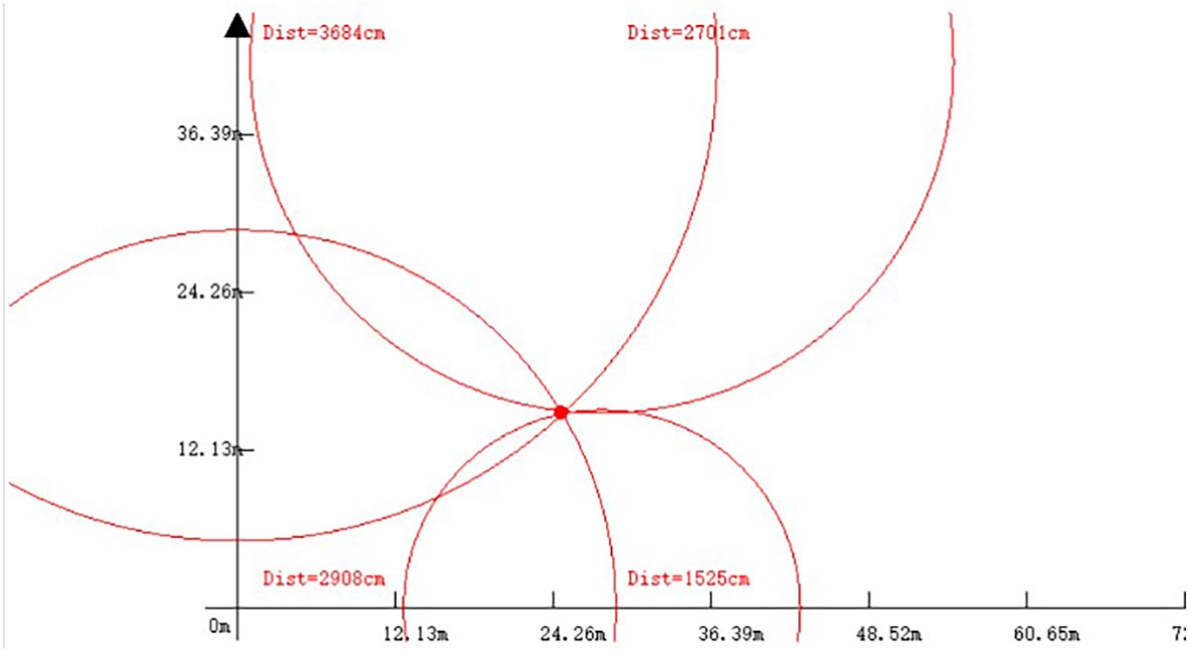
The upper computer positioning circle.

In the experiment, the positioning module used the DWM1000 chip produced by DecaWave, which includes four base stations and one positioning tag. Due to the high transmission rate of UWB technology, positioning data was collected every 20 ms during the experiment, and a total of 1000 samples were collected for analysis. For the indoor static localization experiment using UWB technology, the actual coordinates of the tag were selected as (15,30) for localization detection. The actual localization module used in the experiment is illustrated in Fig.7. The host PC used in the experiment is programmed in $C\#$, with the main base station connected to the computer for sending and receiving positioning data. Additionally, the host PC can display the real-time relative position coordinates of the tags and the distance to each base station. The upper computer positioning circle is depicted in Fig. 8.

### Result analysis

**Figure 9:**
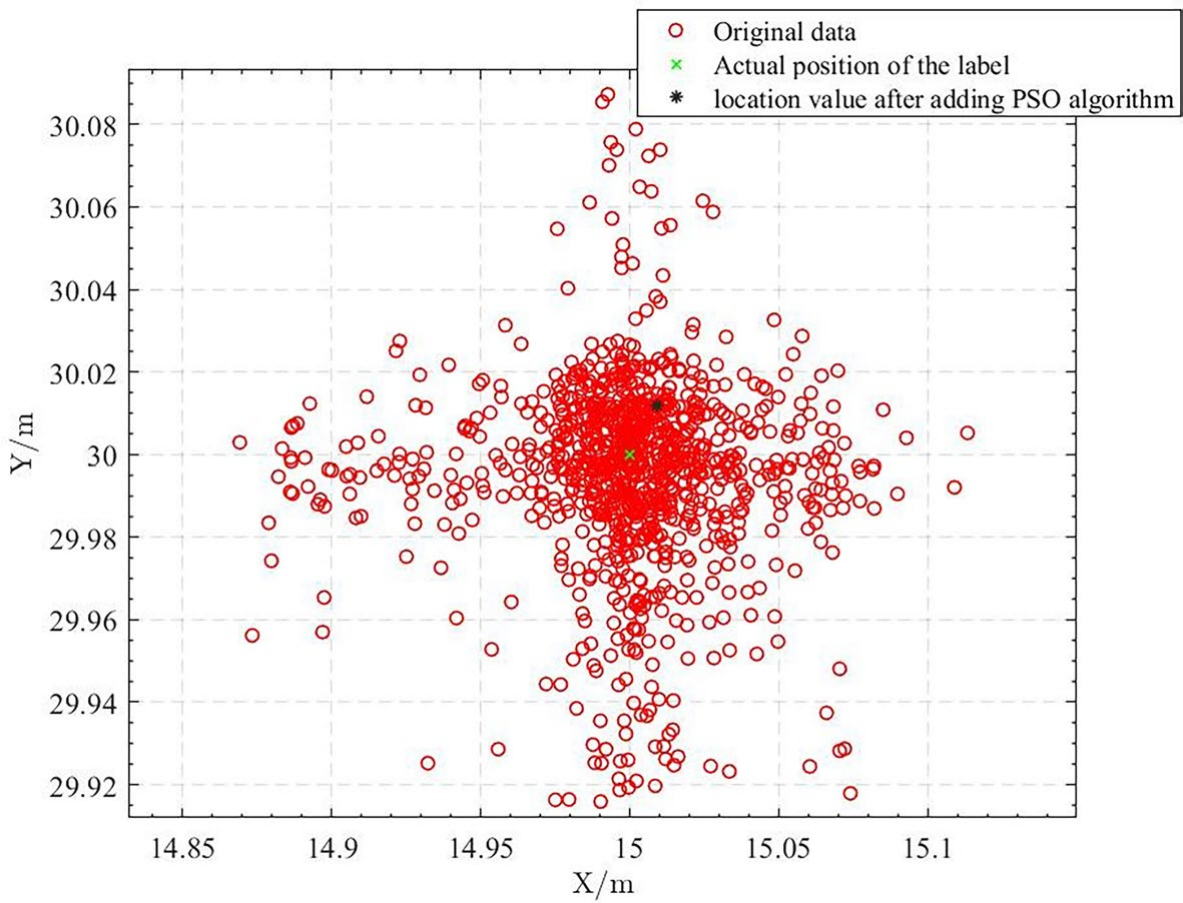
PSO algorithm positioning result.

Figure 9 shows the positioning results processed by the pso algorithm. For processing with only the PSO algorithm, we used the sum of the Euclidean distances between points as the fitness value for the particle swarm algorithm. However, we found a large error between the optimal point and the actual coordinates. The results show that while the PSO algorithm has high application efficiency in indoor localization as a search optimization algorithm, particles can easily fall into local optimal solutions due to the large range of the original data and interference from NLOS errors, leading to unsatisfactory positioning accuracy.

**Figure 10:**
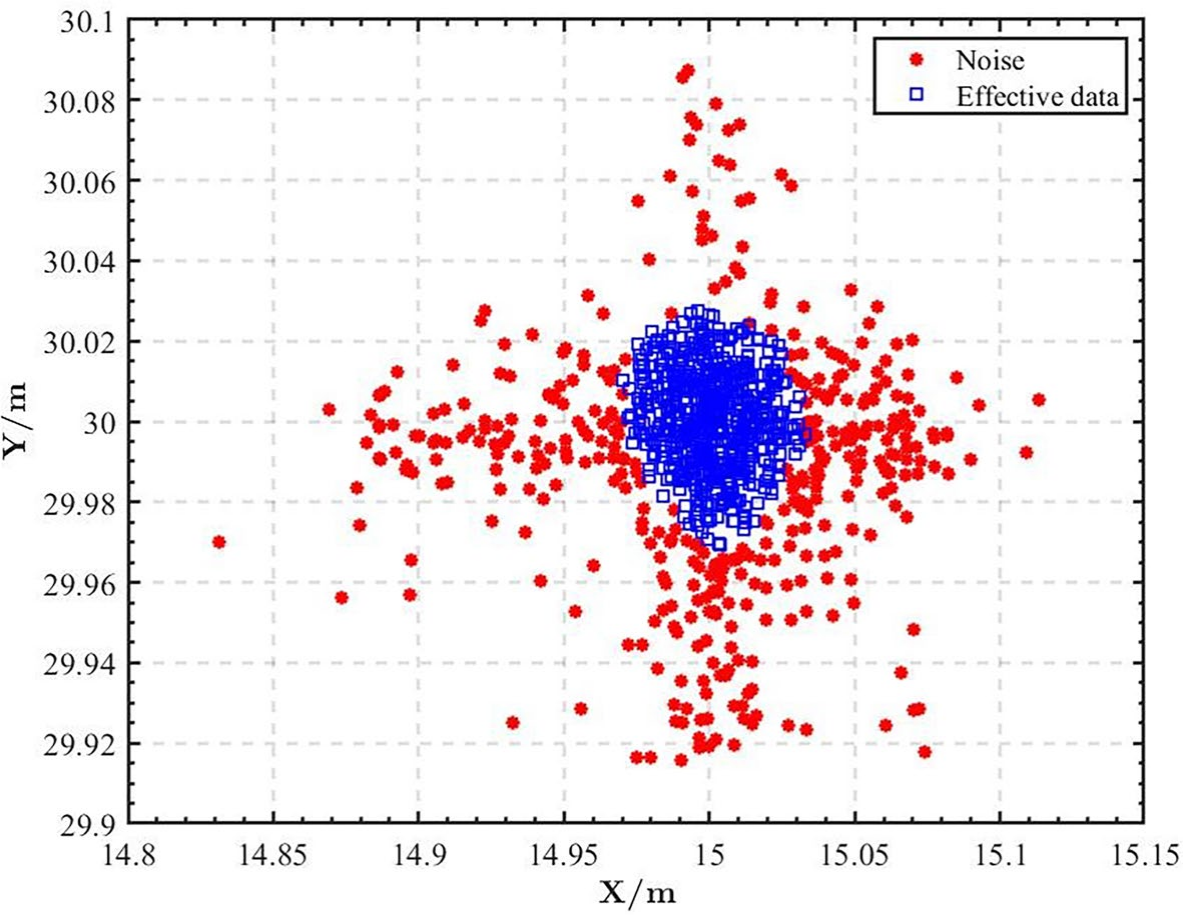
DBSCAN clustering effect diagram.

**Figure 11:**
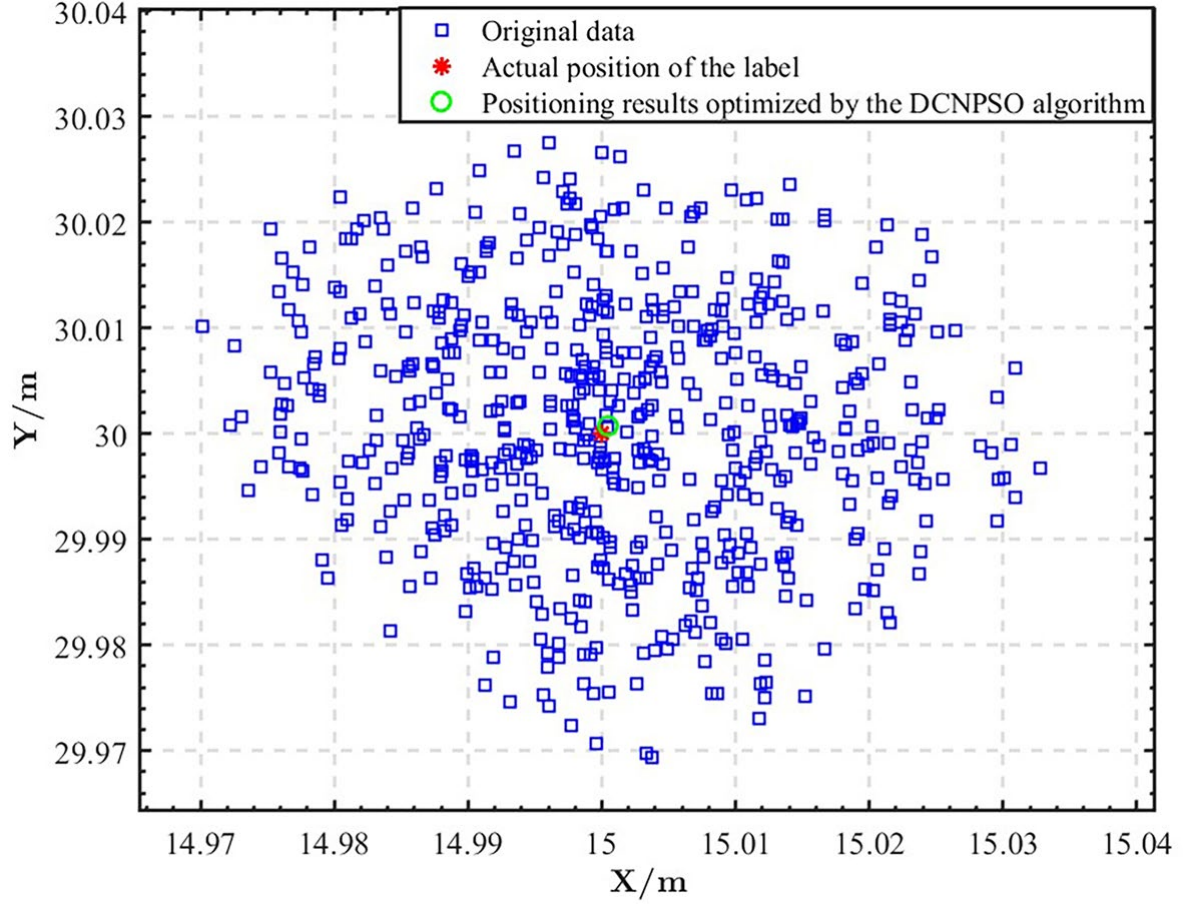
DCNPSO algorithm positioning result.

Next, we will conduct further experiments and analysis to verify the performance of the DCNPSO algorithm in indoor positioning. We utilized the DCNPSO algorithm proposed in this paper to process the same localization data and obtained the results shown in Fig. 10. It is evident that the clustering algorithm automatically divides the raw localization data into two categories. The red points represent noise interference, while the blue points indicate valid data after filtering out the noise. Figure 11 illustrates the test positioning results. We can find that the difference between the positioning results optimized by the DCNPSO algorithm and the actual coordinates is very small. Therefore, the DCNPSO algorithm exhibits promising optimization capabilities in the field of indoor UWB positioning.

Table 1 shows the comparison of the root mean square error of the three algorithms RMSE of the positioning optimized by the DCNPSO algorithm can be controlled below $3 \times {10^{ - 2}}$, as observed after conducting several static positioning tests and comparing the RMSE of the test results. In terms of accuracy, the DCNPSO algorithm has the highest accuracy, 45.25%, and 36.14% higher than the DBSCAN and PSO algorithms, respectively. It is worth noting that the positioning error was stabilized within 3 cm, indicating a significant optimization effect.

**Figure 12:**
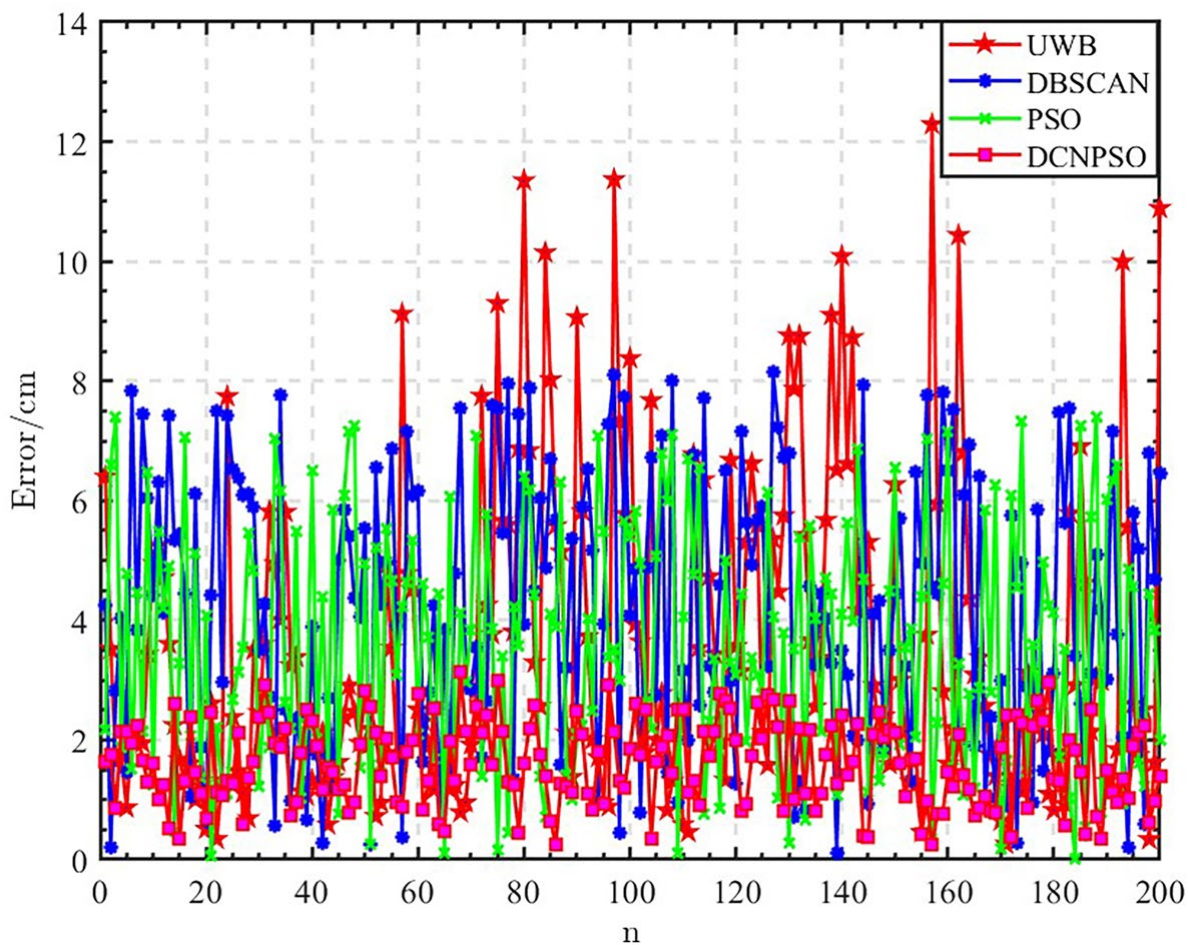
Algorithm error comparison chart.

Figure 12 shows the error comparison of the three algorithms, and it can be found that the DCNPSO algorithm for UWB indoor positioning optimization is better than the remaining two algorithms. However, the DCNPSO algorithm still has defects. Although the DCNPSO algorithm significantly improves positioning accuracy, the complexity of the algorithm is higher than that of the PSO algorithm and the real-time performance is poor.

**Figure 13:**
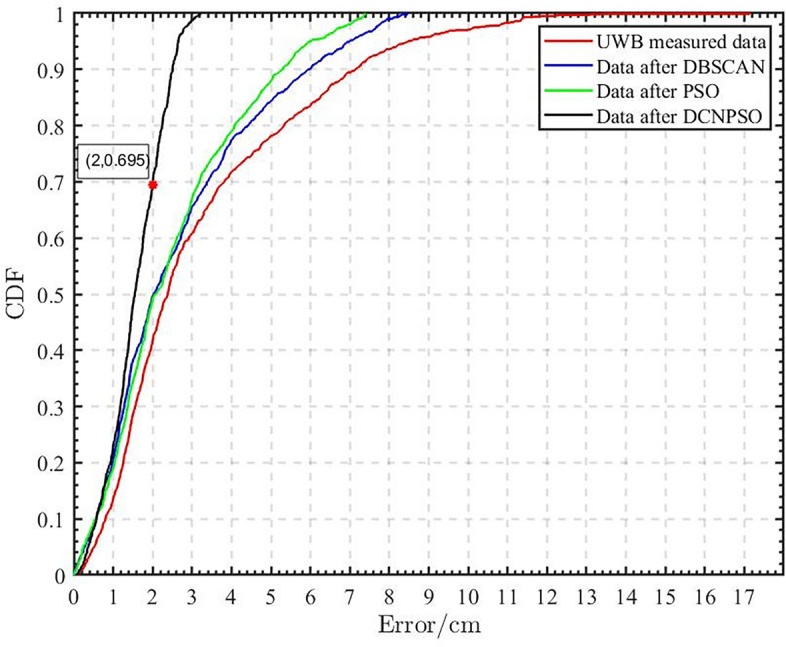
Cumulative distribution of positioning errors.

The cumulative distribution function (CDF) of the localization error is shown in Fig. 13. The horizontal axis represents the positioning error, while the vertical axis represents the probability that the positioning error is less than or equal to the corresponding value on the horizontal axis. The study’s findings reveal that the original UWB positioning data exhibits significant errors in terms of indoor positioning accuracy optimization, with a cumulative distribution of approximately 40% for a 2 cm error. However, the DCNPSO algorithm result shows a 2 cm error cumulative distribution of around 69.5%. This indicates that the proposed DCNPSO algorithm performs exceptionally well in terms of both positioning accuracy and stability. Notably, the error remains highly stable up to 3 cm. In conclusion, the results demonstrate that the combination of the DBSCAN algorithm with the PSO algorithm leads to a substantial increase in positioning accuracy and a significant improvement in positioning stability.

**Table 1:** Comparison of Root Mean Square Error under the three algorithms.

Tag coordinates	PSO algorithm		DBSCAN algorithm		DCNPSO algorithm	
	Locator data	RMSE	Locator data	RMSE	Locator data	RMSE
(5.0,10.0)	(4.923,10.061)	0.0695	(4.961, 10.053)	0.0658	(4.972, 10.019)	0.0156
(10.0,15.0)	(9.921,15.072)	0.0756	(9.931, 15.060)	0.0914	(9.918, 15.023)	0.0239
(15.0,20.0)	(15.054,20.103)	0.0822	(15.087, 20.098)	0.1310	(15.014, 20.022)	0.0184
(20.0,35.0)	(19.953,35.062)	0.0550	(19.938, 35.052)	0.0809	(19.988, 35.014)	0.0130
(25.0,30.0)	(25.127,29.926)	0.1039	(25.052, 29.909)	0.1048	(25.022, 29.977)	0.0220
(30.0,5.0)	(30.070,4.889)	0.0928	(30.067, 4.932)	0.0955	(30.022, 4.974)	0.0241

## Conclusions

To improve the accuracy of UWB indoor positioning and reduce the error caused by NLOS noise, this study proposes an optimization algorithm that combines the DBSCAN algorithm with the PSO algorithm.

The experimental results show that the DCNPSO algorithm significantly reduces the error by 45.25% and 36.14% compared to the DBSCAN and PSO algorithms. The DCNPSO algorithm demonstrates good robustness and high precision in positioning, with a cumulative distribution of 69.5% for errors within 2 cm and error values consistently within 3 cm. These results indicate the strong practical value of the proposed algorithm. These results are significant. The proposed optimized positioning algorithm in this research can be used in the fields of indoor object localization, elderly status monitoring, and item theft prevention. In the next step, we will explore a multi-parallel localization approach to further evaluate the performance of the algorithm and improve its efficiency by reducing the number of iterations, shortening the runtime, and increasing the speed. Further, FPGA hardware acceleration can be used to improve the operation speed of the algorithm.

## Data Availability

These data are generated by real experiments and collected in the experimental area. If necessary, they can be acquired from the corresponding author.
